# Supplementary figures and images for: TUBA1A tubulinopathy mutants disrupt neuron morphogenesis and override XMAP215/Stu2 regulation of microtubule dynamics
Source: eLife. 2022 May 5;11:e76189. doi: 10.7554/eLife.76189 (PMC9236607; doi:10.7554/eLife.76189)

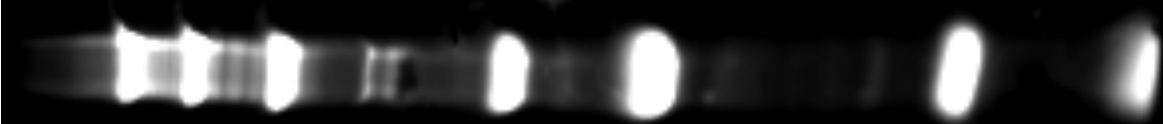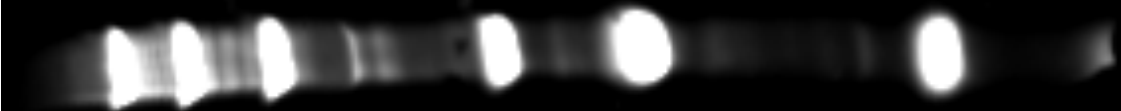

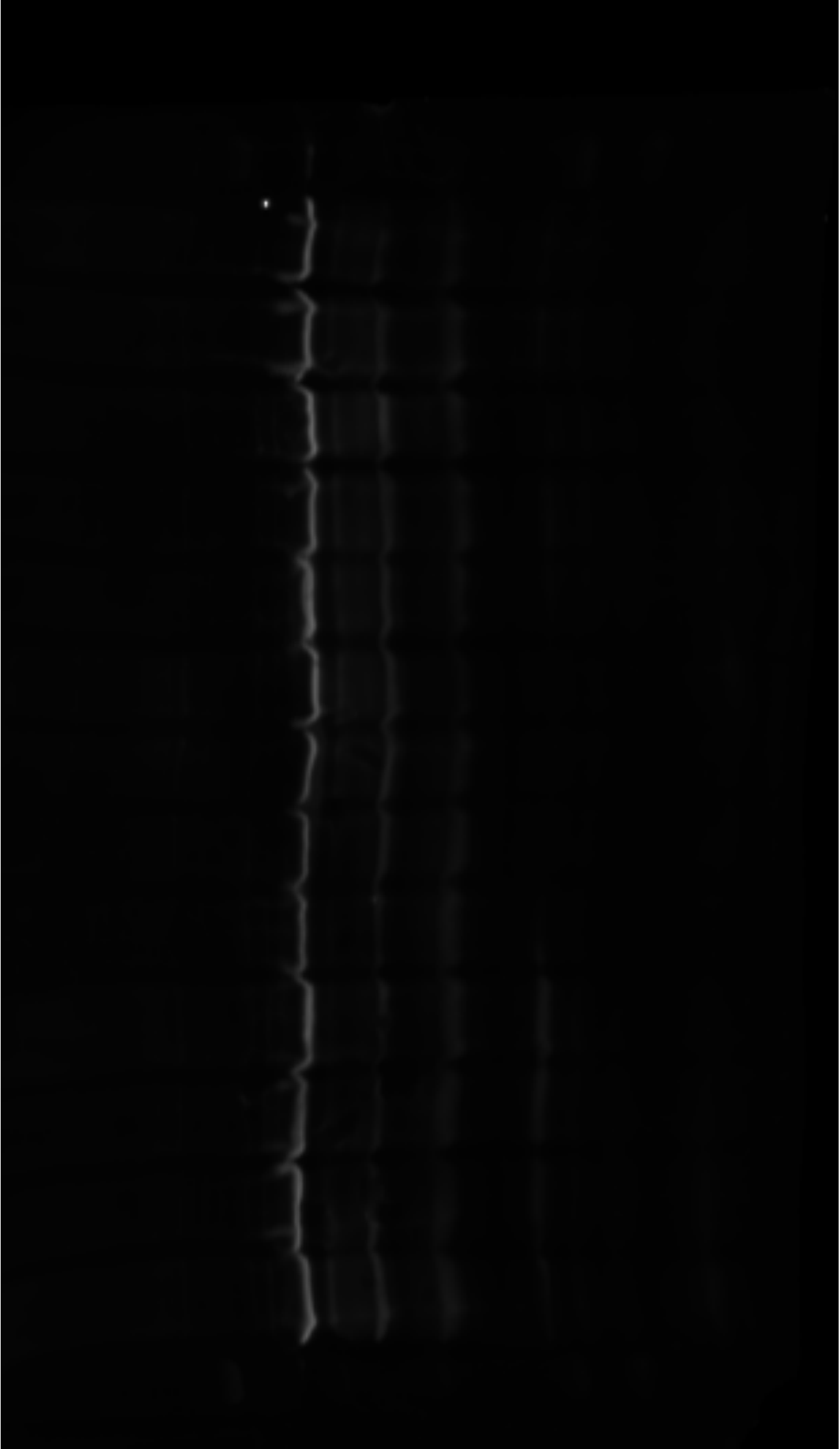

Supplement: Figure 5—source data 4. [file elife-76189-fig5-data4.pdf]

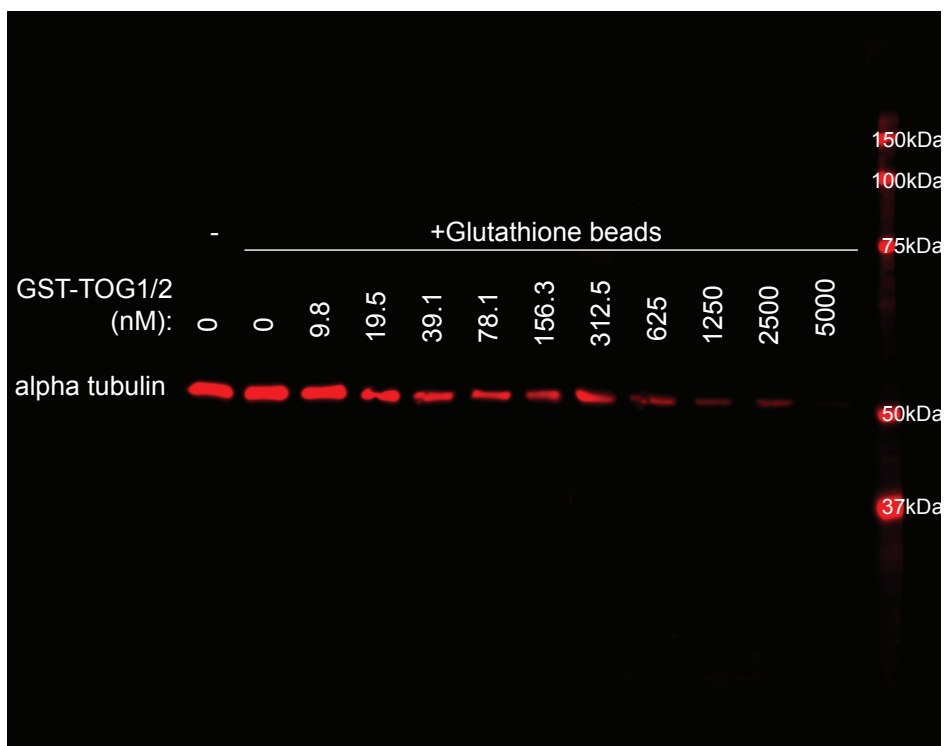

Supplement: Figure 5—figure supplement 1—source data 1. [file elife-76189-fig5-figsupp1-data1.pdf]

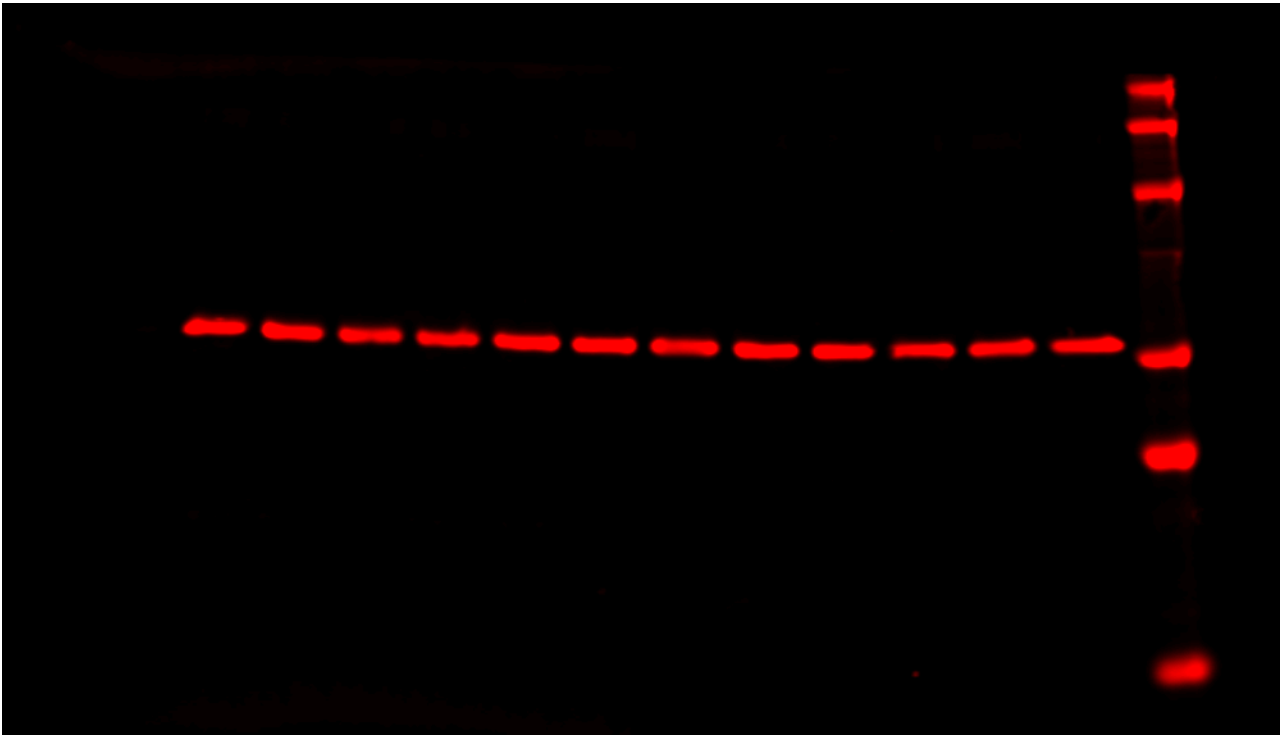

Supplement: Figure 5—figure supplement 1—source data 3. [file elife-76189-fig5-figsupp1-data3.pdf]

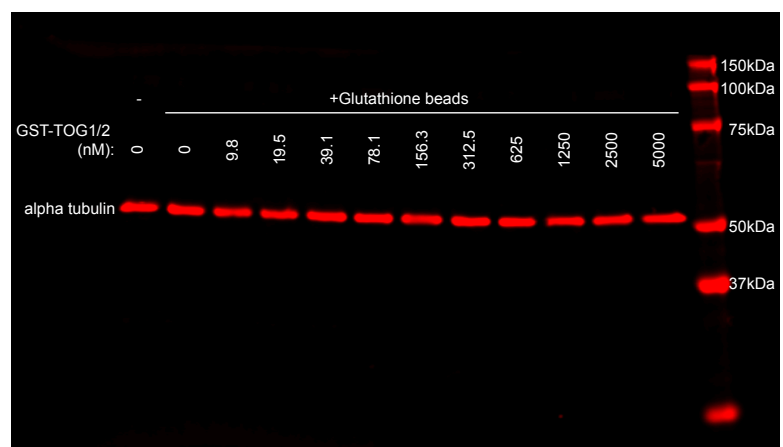

Supplement: Figure 5—figure supplement 1—source data 4. [file elife-76189-fig5-figsupp1-data4.pdf]

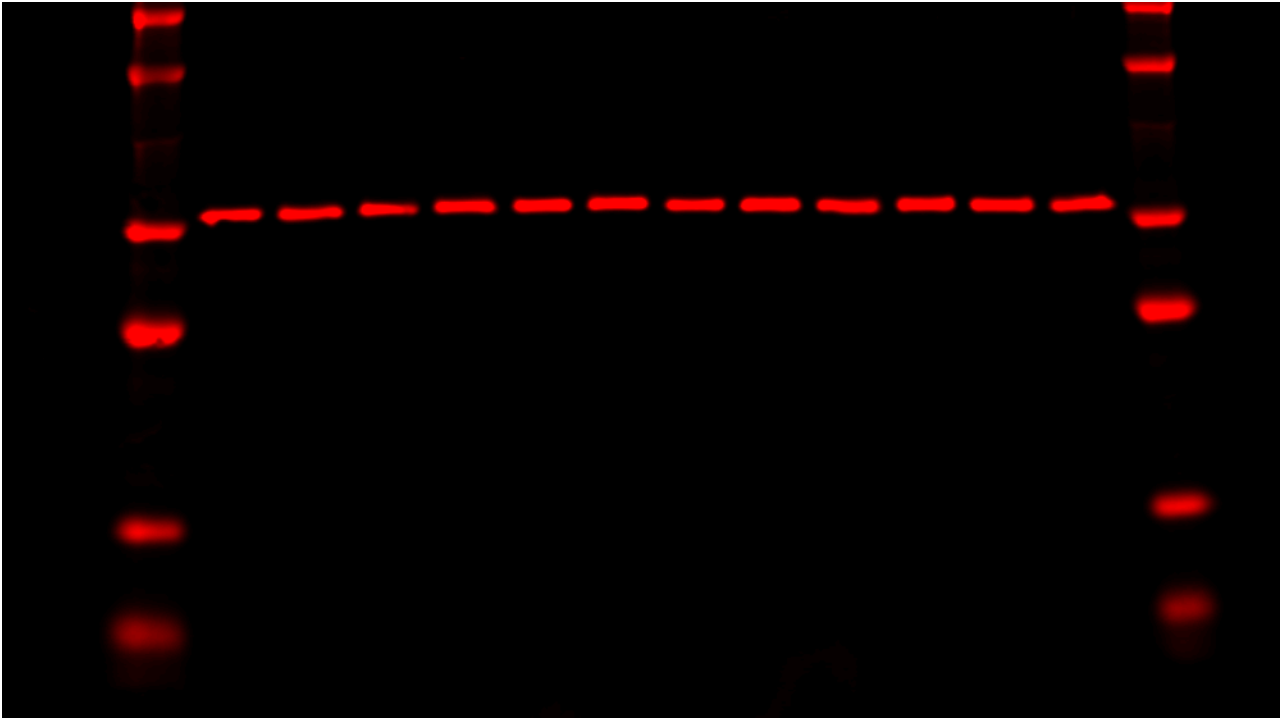

Supplement: Figure 5—figure supplement 1—source data 5. [file elife-76189-fig5-figsupp1-data5.pdf]

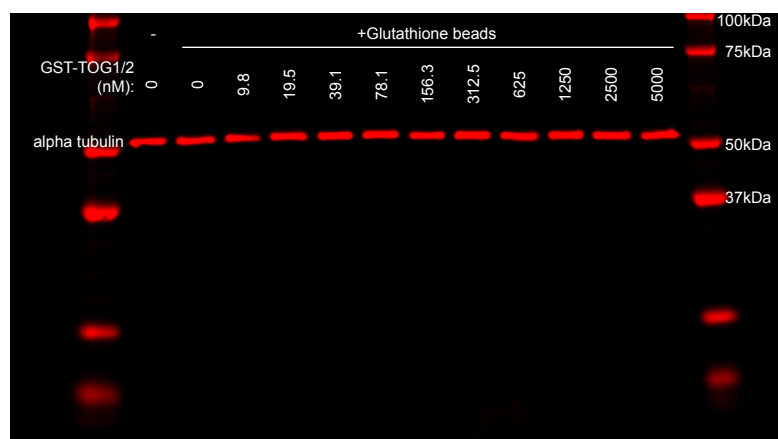

Supplement: Figure 5—figure supplement 1—source data 6. [file elife-76189-fig5-figsupp1-data6.pdf]

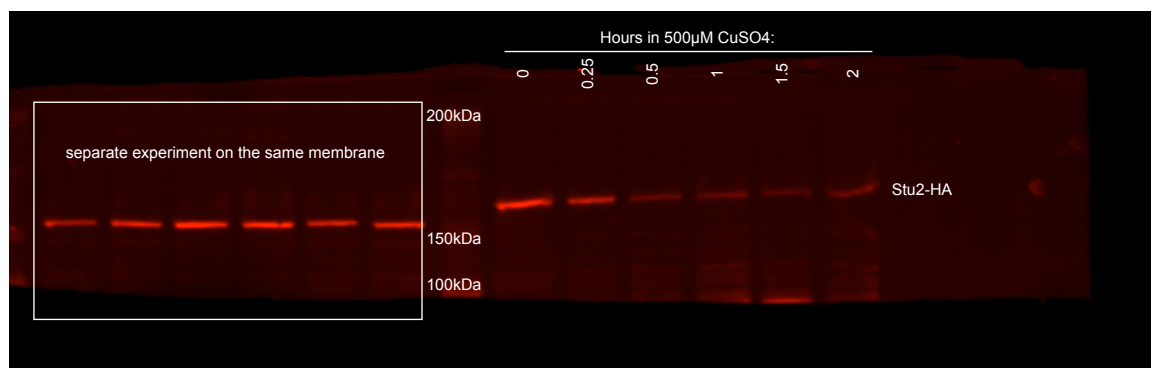

Supplement: Figure 5—figure supplement 1—source data 8. [file elife-76189-fig5-figsupp1-data8.pdf]

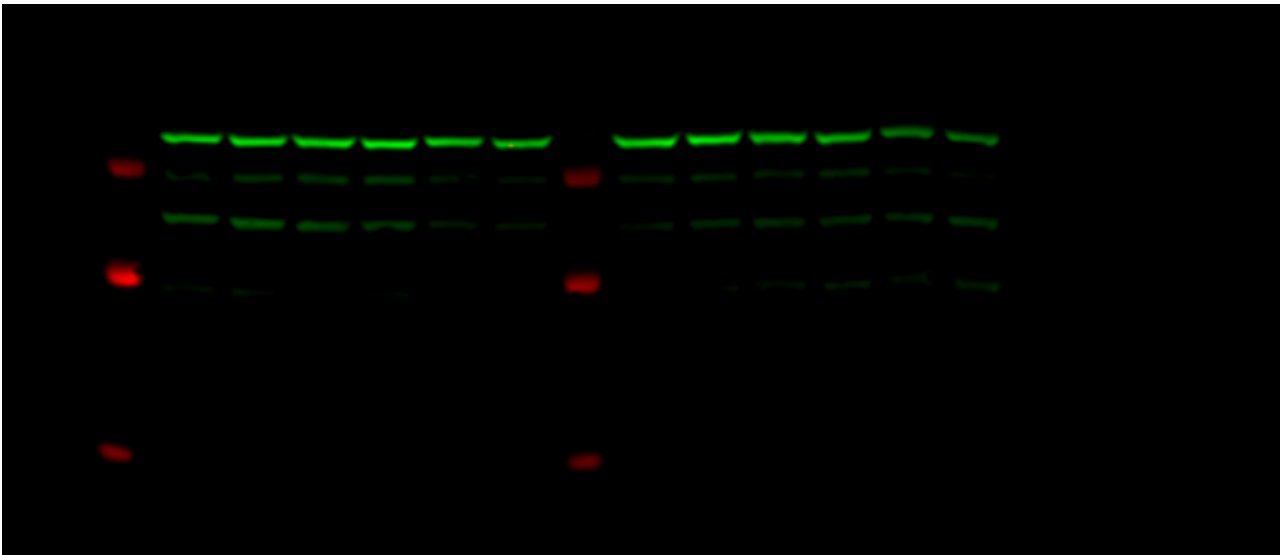

Supplement: Figure 5—figure supplement 1—source data 9. [file elife-76189-fig5-figsupp1-data9.pdf]

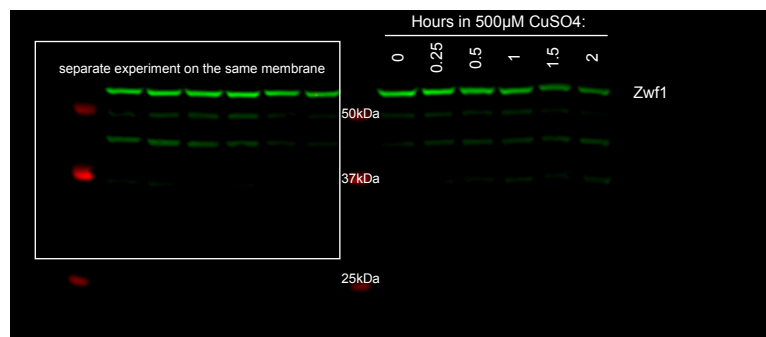

Supplement: Figure 5—figure supplement 1—source data 10. [file elife-76189-fig5-figsupp1-data10.pdf]
