# Supplementary material for: TUBA1A tubulinopathy mutants disrupt neuron morphogenesis and override XMAP215/Stu2 regulation of microtubule dynamics
Source: eLife. 2022 May 5;11:e76189. doi: 10.7554/eLife.76189 (PMC9236607; doi:10.7554/eLife.76189)

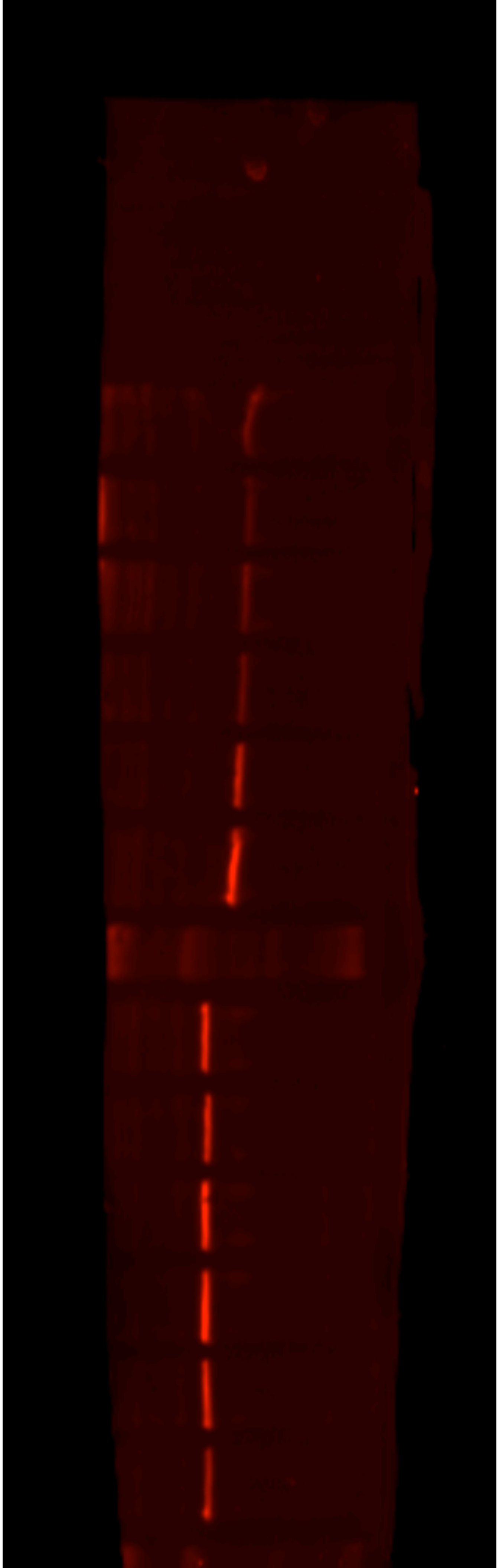

Supplement: Figure 5—figure supplement 1—source data 7. [file elife-76189-fig5-figsupp1-data7.pdf]
