# Supplementary material for: TUBA1A tubulinopathy mutants disrupt neuron morphogenesis and override XMAP215/Stu2 regulation of microtubule dynamics
Source: eLife. 2022 May 5;11:e76189. doi: 10.7554/eLife.76189 (PMC9236607; doi:10.7554/eLife.76189)
Supplement: Figure 5—source data 5. [file elife-76189-fig5-data5.pdf]

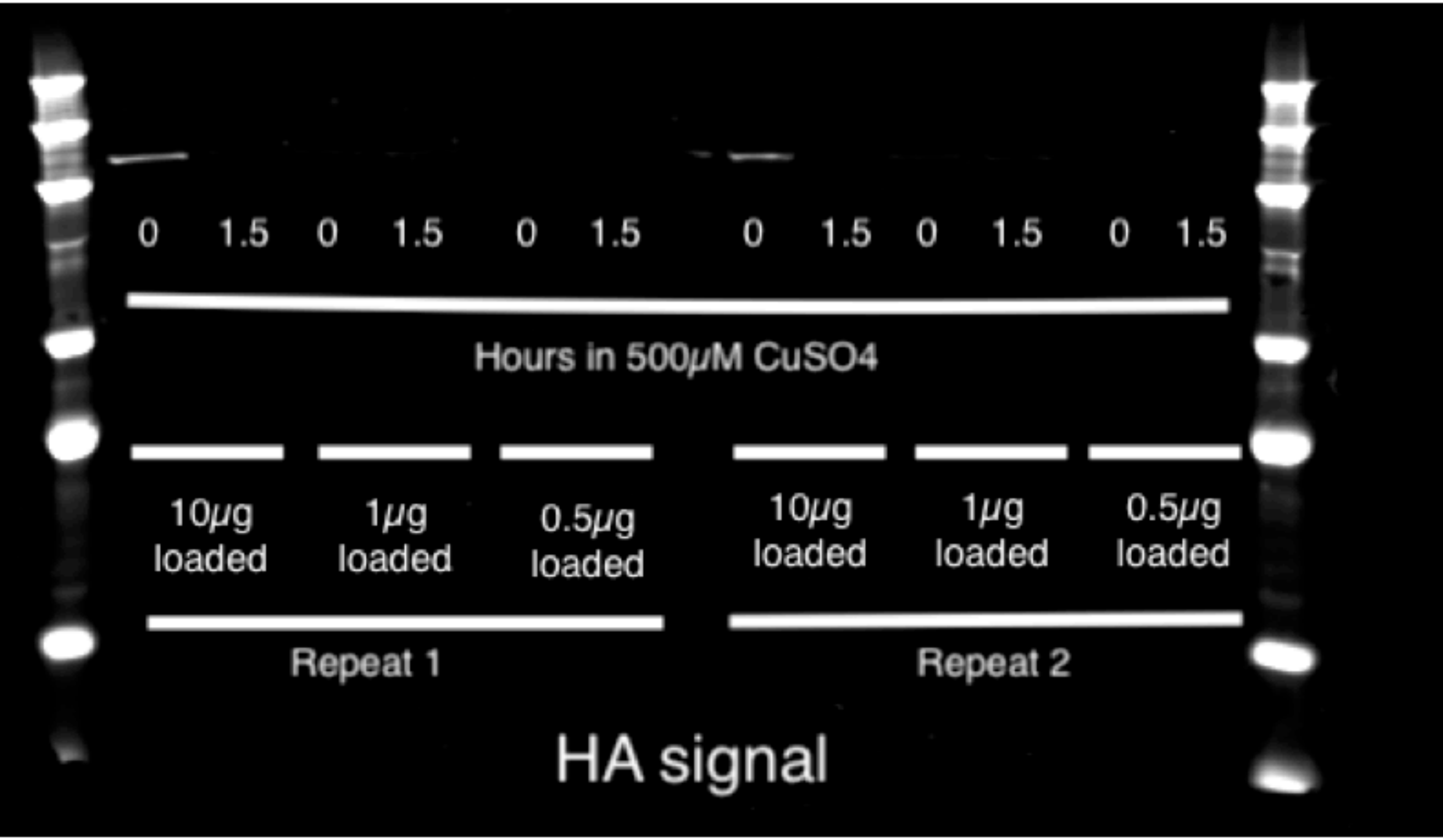
0 1.5 0 1.5 0 1.5 0 1.5 0 1.5 0 1.5
Hours in 500μM CuSO4
10μg loaded
1μg loaded
0.5μg loaded
10μg loaded
1μg loaded
0.5μg loaded
Repeat 1
Repeat 2
HA signal

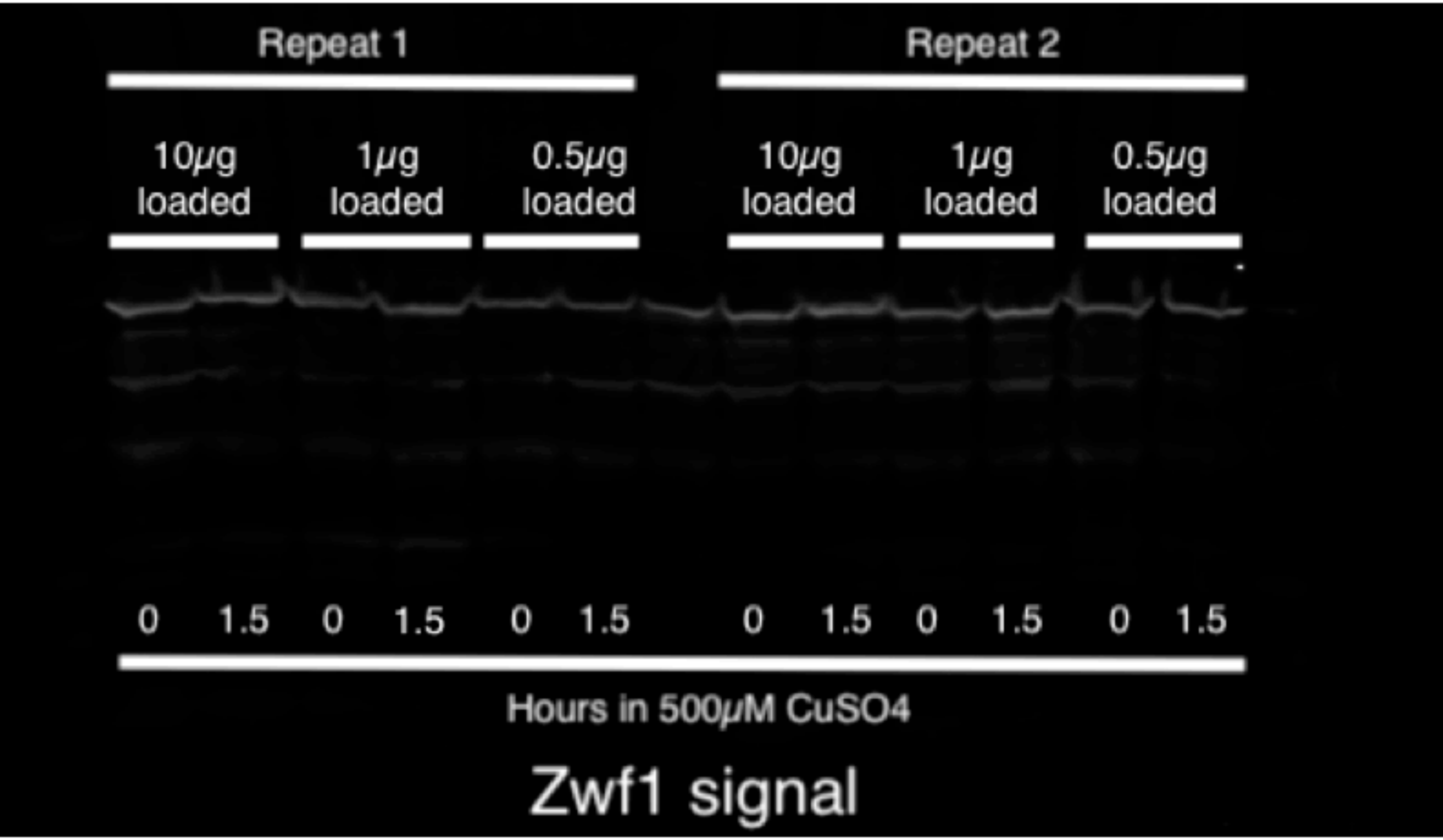
Repeat 1
Repeat 2
10μg loaded
1μg loaded
0.5μg loaded
10μg loaded
1μg loaded
0.5μg loaded
0 1.5 0 1.5 0 1.5 0 1.5 0 1.5 0 1.5
Hours in 500μM CuSO4
Zwf1 signal
